# The complete mitochondrial genome of electric catfish *Malapterurus electricus* and its phylogeny

**DOI:** 10.1080/23802359.2022.2054381

**Published:** 2022-03-22

**Authors:** Yongxin Li, Hui Jiang

**Affiliations:** a School of Ecology and Environment, Northwestern Polytechnical University, Xi’an, China; b College of Life Science, Hainan Normal University, Haikou, China

**Keywords:** *Malapterurus electricus*, electric catfish, mitochondrial genome, phylogeny, next-generation sequencing

## Abstract

Electric catfishes evolved the substantial electric organ that can instantly release powerful high-voltage electricity. To better study the phylogenetic position of the electric fish (*Malapterurus electricus*) in catfishes, in this study, we presented the complete mitochondrial genome of *M. electricus* assembled by the next-generation sequencing data. The mitogenome has 16,504 bp and contains 13 protein-coding genes (PCGs), 22 transfer RNA genes, two ribosomal RNA genes, an L-strand replication origin (OL), and a control region (D-loop). The overall base composition is A 31.08%, C 27.54%, G 14.81%, and T 26.57%. Phylogenetic analysis based on 13 PCGs of 43 species from Siluriformes showed *M.electricus* belonging to the Malapteruridae displayed a close relationship with Siluridae. Taken together, the complete mitochondrial genome of *M. electricus* would be beneficial for the study of the phylogenetic relationship of Siluriformes.

*Malapterurus electricus* belongs to Malapterurus; Malapteruridae; Siluriformes, is mainly distributed in the freshwater basins of tropical Africa (Carl 2007; Diouf et al. 2020). The main external morphological characteristics of *M. electricus* include: (1) cylindrical body; (2) scaleless body surface; (3) three pairs of whiskers around its mouth; (4) without dorsal fin (Howes 1985; Norris 2002; Welzel and Schuster 2021). However, unlike other catfishes, electric catfishes evolved the substantial electric organ that can instantly release powerful high-voltage electricity for predation and defense (Janetzko et al. 1987). Although many detailed anatomy and discharge waveform of electric organ has been studied in electric catfish (Volknandt and Zimmermann 1986; Schikorski et al. 1994a, 1994b), the special genetic evolutionary relationship of *M. electricus* in Siluriformes analyzed by the mitochondrial genome is still scarce. In this study, we successfully assembled and annotated the complete mitochondrial genome of *M. electricus* using the next-generation sequencing data.

The *M. electricus* sample was acquired from Xiamen (118.0995 E, 24.4685 N) and stored in a refrigerator of −80 °C at School of Ecology and Environment, Northwestern Polytechnical University (Yongxin Li; yxli28science@sina.com) under the voucher number 20180901DN01. The species was identified based on morphologic features and *COX1* gene (GenBank accession No. EU179811.1). All procedures were approved by the Medical and Animal Experimental Ethics Committee of Northwestern Polytechnical University and followed the guidelines for the care and use of laboratory animals. The whole genome sequencing data used in this study was produced on the sequencing platform of NovaSeq 6000 (Illumina, USA). The complete mitochondrial genome of *M. electricus* (Genbank accession No.OL802922) was assembled by the MitoZ software (Meng et al. 2019) with default parameters, which has 16,504 bp in total with overall nucleotide composition of 31.08% A, 27.54% C, 14.81% G, and 26.57% T.

The annotation of the *M. electricus* mitochondrial genome was conducted by the MITOS Web Server (Bernt et al. 2013) and tRNAscan-SE Search Server (Chan and Lowe 2019). The gene arrangement and transcriptional orientation in *M. electricus* are similar to most teleosts (Luo et al. 2017; Prakhongcheep et al. 2017; Sato et al. 2021) with containing 13 protein-coding genes (PCGs), 22 tRNA genes, two rRNA genes (12S rRNA and 16S rRNA), an L-strand replication origin (OL), and a control region (D-loop). Except for *COX1* used GTG as an initiation codon, the other 12 PCGs started with the conventional initiation codon of ATG. For termination codon, six PCGs (*ATP6*, *ATP8*, *COX1*, *ND4L*, *ND5* and *ND6*) ended with TAA, three PCGs (*ND1*, *ND2* and *ND3*) with TAG, and the stop codons of *COX2*, *ND4* and *CYTB* are an incomplete T–, while only *COX3* with TA-. The total length of 13 PCGs is 11,373 bp, which accounting for 68.91% of the complete mitogenome. Besides, all tRNAs were predicted successfully to fold into a classical cloverleaf structure except trnS-GCT, with their length ranging from 67 bp (trnC-GCA) to 75 bp (trnL-TAA). 12S rRNA was identified located between trnF-GAA and trnV-TAC with 954 bp in length, and 16S rRNA located between trnV-TAC and trnL-TAA with 1662 bp. Furthermore, the lengths of D-loop located between trnP-TGG and trnF-GAA and OL located between trnN-GTT and trnC-GCA were 866 bp and 29 bp, respectively. Compared with the mitochondrial genome which is already present in GeneBank database (https://www.ncbi.nlm.nih.gov/nuccore/AP012016.1), our mitochondrial genome annotation information is more comprehensive and detailed. Specifically, (1) we annotated 22 tRNAs in more detail, and gave all the annotation description of anti-codon; (2) two non-coding regions are successfully annotated and explained by us.

In order to systematically analyze the phylogenetic relationship of *M. electricus* in Siluriformes, the mitogenomes of 43 species (belongs to 25 families) from Siluriformes and one outgroup species (*Danio rerio*) were downloaded from GenBank nucleotide sequences database in NCBI. The multiple sequence alignment was performed by the ClustalW software (Thompson et al. 1994). The phylogenetic relationship was constructed based on 13 PCGs using the neighbor joining (NJ) methods with 10,000 bootstrap replications by MEGA7 (Kumar et al. 2016). The result of phylogenetic analysis indicated that *M. electricus* belonging to Malapteruridae displayed a close relationship with Siluridae (Figure 1). We expect that these results would be beneficial for the study of the phylogenetic relationship of Siluriformes.

**Figure 1.**
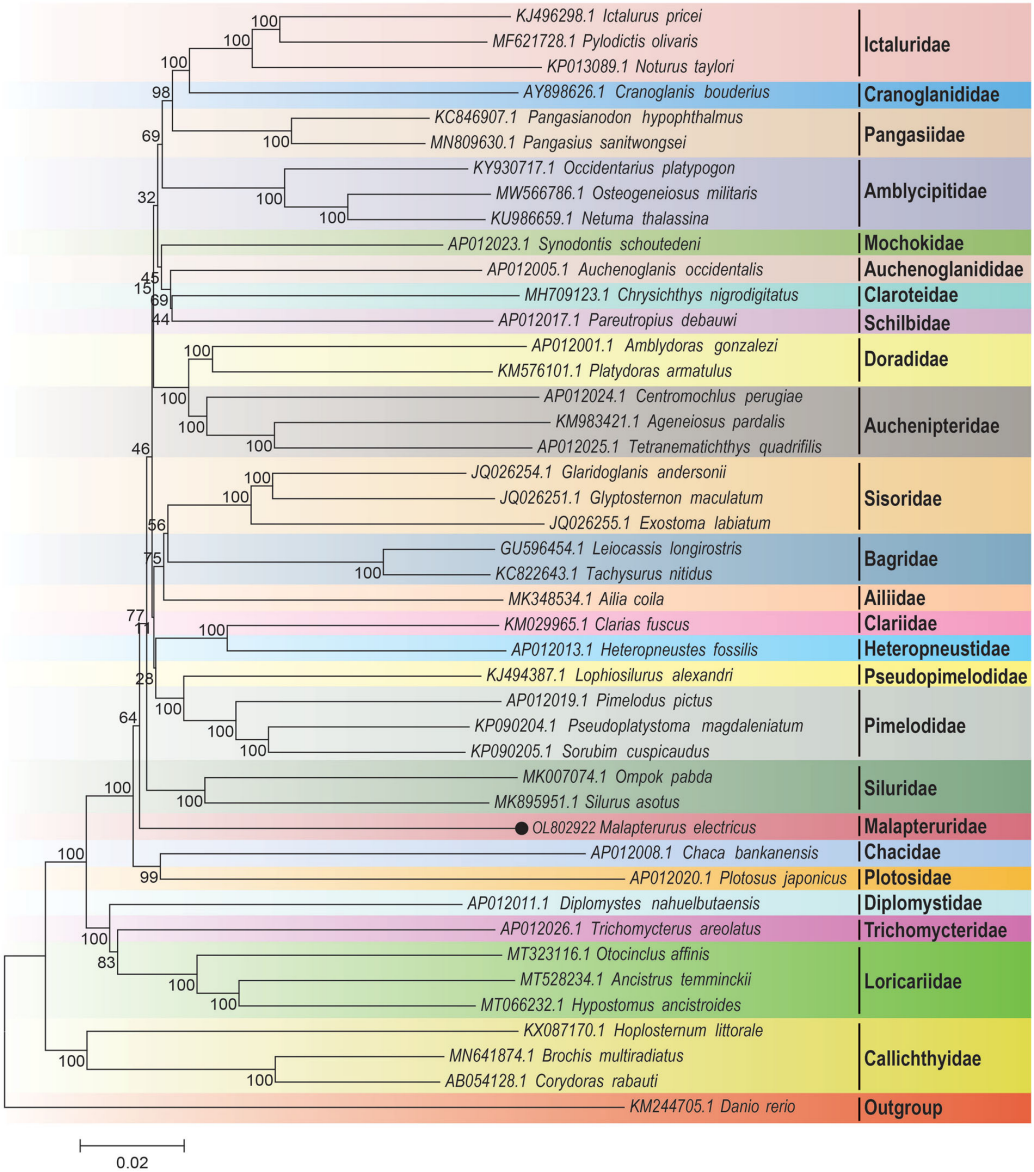
Neighbor-Joining tree based on the 13 protein-coding genes of 43 Siluriformes species and one outgroup species. The number at each node is the bootstrap probability. The number before the species name is the accession number in GenBank. The dark spot indicates the studied species.

## Data Availability

The mitochondrial genome data that supports the findings of this study are openly available in GenBank of NCBI at https://www.ncbi.nlm.nih.gov under the accession number OL802922. The associated BioProject, SRA, and BioSample numbers are PRJNA789410, SRR17272310, and SAMN24108793, respectively.
